# Community-level factors associated with the use of facility-based delivery assistance in Uganda: a multilevel analysis

**DOI:** 10.1186/s12884-020-2851-0

**Published:** 2020-04-03

**Authors:** Angela E. Micah, David R. Hotchkiss

**Affiliations:** 1 Institute for Health Metrics and Evaluation, Seattle, USA; 2 Tulane University School of Public Health and Tropical Medicine, New Orleans, USA

**Keywords:** Facility-based delivery, Community level factors, Uganda

## Abstract

**Background:**

In low- and middle-income countries, the proportion of pregnant women who use health facilities for delivery remains low. Although delivering in a health facility with skilled health providers can make the critical difference between survival and death for both mother and child, in 2016, more than 25% of pregnant women did not deliver in a health facility in Uganda. This study examines the association of contextual factors measured at the community-level with use of facility-based delivery in Uganda, after controlling for household and individual-level factors.

**Methods:**

Pooled household level data of 3310 observations of women who gave birth in the last five years is linked to community level data from the Uganda National Panel Survey (UNPS). A multilevel model that adequately accounted for the clustered nature of the data and the binary outcome of whether or not the woman delivered in a health facility was estimated.

**Results:**

The study findings show a positive association at the county level between place of delivery, education and access to health services, and a negative association between place of delivery and poverty. Individuals living in communities with a high level of education amongst the household heads were 1.67 times (95% Confidence Interval: 1.07–2.61) more likely to have had a facility-based delivery compared to women living in communities where household heads did not have high levels of education. Women who lived in counties with a short travel time (less than 33 min) were 1.66 times (95% CI: 1.11–2.48) more likely to have had a facility-based delivery compared to women who lived in counties with longer travel time to any health facility. Women living in poor counties were only 0.64 times (95% CI: 0.42–0.97) as likely to have delivered in a health facility compared to pregnant women from communities with more affluent individuals.

**Conclusions:**

The findings on household head’s education, community economic status and travel time to a health facility are useful for defining the attributes for targeting and developing relevant nation-wide community-level health promotion campaigns. However, limited evidence was found in broad support of the role of community level factors.

## Background

According to the World Health Organization, approximately 830 women die every day due to pregnancy related complications or childbirth. In 2015 alone, an estimated 303,000 women died worldwide during and after pregnancy and childbirth [1]. Almost all of these women live in low- and middle- income countries. In Uganda, 5700 pregnant women died in 2015 and an estimated 34,151 children were still born [1, 2]. Most of these deaths could have been prevented. This is because these deaths arise from pregnancy related complications such as unsafe abortions, high blood pressure during pregnancy, and delivery complications that may be adequately managed in a health facility by a skilled provider [3].

Thaddeus and Maine (1994) in their review of the factors that contribute to maternal mortality highlighted three phases of delay in accessing quality care for delivery [4]. First, delays related to seeking care. Second, delays related to reaching care and lastly delays related to receiving care once pregnant women arrived at the facility. More recently, the Lancet published a series of six papers that detailed the state of maternal knowledge – its epidemiology, progress and challenges [5–9]. While some progress has been made globally in reducing maternal mortality rates, that progress is varied and is often contingent on geographic location and socioeconomic status. Some pregnant women are receiving too little care too late while others receive too much care too soon [9].

The existing literature on the determinants of facility-based delivery is extensive [10–20]. Results from this literature point to various cultural, economic and social reasons that facilitate or deter the use of health facilities for delivery. Economic barriers such as the costs of delivery, the hidden costs from having to purchase delivery supplies in some instances and other additional costs such as for transportation have been found to act as barriers to facility-based deliveries [16, 20–22]. In some settings, cultural beliefs regarding birthing as a natural process discourages institutional deliveries unless there is a complication associated with the delivery [21, 23]. Furthermore, in some societies, the perceived quality of care from traditional birth attendants may be higher than in a facility because women are treated respectfully in familiar environments and with more privacy [24, 25]. Attendant staff shortages and a lack of infrastructure in health facilities can also deepen perceptions of low quality of care delivered in the health facilities and act as deterrents [23, 26].

While delivering in a health facility with skilled health providers can make the critical difference between survival and death for both mother and child, in low- and middle-income countries, the proportion of pregnant women who use health facilities for delivery still lags behind the uptake of antenatal care services despite increases in recent years due to a concerted global effort to increase deliveries in facilities and reduce maternal mortality. For instance, while deliveries in health facilities in Uganda have increased from 57% in 2011 to 73% in 2016, 97% of these pregnant women attended antenatal care at least once in 2016 [27, 28]. The high antenatal care attendance levels suggest that women are able to engage initially with the health system but are constrained from utilizing facility-based delivery services perhaps by other contextual factors such as impersonal treatment or mistreatment by health staff associated with health facility deliveries.

Qualitative studies have been conducted to understand why pregnant women in low- and middle-income countries continue to deliver at home instead of in health facilities [11, 29–31]. Key findings from this research point to cultural norms that encourage and support home delivery, poor treatment of women by health workers, resource constraints and physical inaccessibility. In a study in northern Uganda that examined the factors underlying the antenatal care versus facility delivery gap, the results showed that primary barriers to subsequent delivery at a health facility after an antenatal visit included fear of maltreatment by health workers, socio-cultural and gender issues, lack of spousal support, challenges related to transportation and poverty [32]. Various empirical studies on Uganda have corroborated the socioeconomic and infrastructural related challenges to accessing facility-based deliveries but fewer empirical studies have investigated the complex role of socio-cultural norms and the influence of others in the decision making process of where a pregnant woman gives birth and in particular used cluster-level measures of social norms.

Given the variations across Uganda in relation to service availability and other cultural norms, the available body of evidence on determinants of facility-based delivery in Uganda is deficient in two ways. First, it is limited in terms of the applicability of the findings to the entire populace. Second, the quantitative evidence has yet to fully incorporate the role of the characteristics of the communities in which these women live in their decision to deliver in a health facility. This study tackles the two limitations highlighted in the available literature on facility-based delivery in Uganda by examining community level factors associated with the place of delivery on a pooled dataset of a nationally representative sample of women who had recently given birth in Uganda using a model that accounts for the clustered nature of the data. Incorporating community related factors is important because child birth is a communal event in this setting and so the preferences of the woman’s community play a role in the woman’s decision making related to this life event.

The study aims to examine the contextual factors that are associated with health facility delivery in Uganda. In the African context, local beliefs about the causes of disease and the myriad of decision makers in the community play complex but important roles in understanding decision-making on location of delivery [33]. It is therefore important to quantify the role of community level factors because it helps to identify the health risks associated with particular social structures and community ecologies [13]. Additionally, understanding how the characteristics, experiences and behaviors of other people influence the decision making and subsequent behavior patterns of pregnant women will assist with the development of appropriate interventions and policies at the community level. Furthermore, it is particularly important to understand the role of context on the place of delivery for pregnant Ugandan women as previous studies based on sub-national samples highlight different level of importance for the factors that influence the choice of delivery location.

## Methods

The data used in this study is from the Uganda National Panel Survey (UNPS) [34–36]. The UNPS is based on a nationally representative sample of households. The survey tracks respondents from 3123 households selected from 322 enumeration areas used as part of the last Uganda National Household Survey 2005/06. Enumeration areas (EA) are included from Kampala District, the Central region with the exception of Kampala District, the Eastern region, the Northern region and the Western Region. A pooled cross sectional design based upon three rounds of data (2009/10, 2010/11, 2011/12) is employed in this study.

During the household listing component of the survey, women of reproductive age - between 15 through 49 years – are identified to also complete a separate women’s module. The women’s module collects data on reproductive health such as contraceptive knowledge and use, birth history and delivery-related health seeking behavior. Additionally, data from the community module on the availability and quality of health services is linked to the women’s data for this analysis. The community survey gathered data on communities in the EAs sampled in the survey. Key informants such as health facility heads completed the health section in the community surveys. The cluster level in this analysis is the county. The analysis sample included 141 counties with 25 to 311 respondents per county. The primary sampling unit, the enumeration area, was not used as a cluster unit because the number of women who had given birth in the previous five years within each unit was not large enough to generate unbiased estimates at that level. The data for the analysis was pooled to overcome this limitation. The county is a local government administrative unit and has an average population of about forty-six thousand [37]. The county level is relevant and appropriate for this analysis because it is the level of the local government at which citizens have a representation in parliament to advocate for their communal interests at the national level. In multilevel analysis of samples for which there are 25 observations per cluster and the intra-cluster correlation is less than or equal to 0.20 bias in estimates is not a problem [38]. Therefore, given the distribution of the analysis sample, bias in the estimates is not a problem in this study. Table 1 below details how the sample was developed.

**Table 1:** Sample selection criteria

Sample selection criteria	No. of observations dropped from sample	No. of women round observations remaining in sample
# of women in pooled sample		10,511
Missing county observations	664	9847
Women who did not give birth in last five years	6418	3429
Missing outcome (facility delivery) variable	119	3310

The dependent variable is a binary indicator of whether or not the respondent’s last birth delivery took place in a health facility. All births that took place in the following locations: government hospital, government health center, government health post, other government facility, private hospital, private clinic, and other private medical facility are classified as having occurred in a health facility whereas births that took place in the woman’s own home, at a traditional birth attendant’s home and another home is classified as not having occurred in a health facility. Thirty-nine percent of women in the sample delivered outside a health facility. Approximately, 3.4% of the place of delivery responses was missing. Of this 0.02% did not know or recall the location of their most recent delivery and the remaining had no responses.

Table 2 below describes the variables used in this analysis. The key explanatory variables of interest are the derived county level variables. These variables are measured as proxies for the prevalence of norms on family size, female autonomy, educational attainment, infant deaths, and neighborhood poverty in each county. The average distance to any health facility and the quality of the services provided are included as measures of service access and perceptions of quality. The data for the last two variables were gathered based on the lowest administrative council area, LC1. In each enumeration area one LC1 was randomly selected and from which data was collected. Ideally, data on the specific facilities available in each of the communities represented in the women’s sample will more accurately reflect the health facility market based on which their decision was made. The LC1 based health service quality variables had many missing observations (6%) and the educational attainment variables have between 8 to 11% missing observations. The preferred multilevel models are rerun excluding these variables to show whether the results are affected by the missingness in these variables.

**Table 2:** Description of independent variables used in the analysis

Variable	Operational Definition
*County Level*	
Rural	Binary variable where 1 indicates woman lives in a rural area, 0 otherwise
Infant death	Binary variable where 1 indicates woman lives in a county in which the proportion of infant death is more than 0.10 (the national mean based on the sample), 0 otherwise
Woman’s Education	Binary variable where 1 indicates woman lives in a county in which the proportion of women with more than a primary education is more than 0.10 (the national median based on the sample), 0 otherwise
Household Head’s Education	Binary variable where 1 indicates household head lives in a county in which the proportion of household heads with more than a primary education is more than 0.28 (the national median based on the sample), 0 otherwise
Poverty concentration	Binary variable where 1 indicates woman lives in a county in which the proportion of women living in households who are below the poverty line in 05/06 prices is more than 0.22 (the national median based on the sample), 0 otherwise
Large family norm	Binary variable where 1 indicates woman lives in a county in which the proportion of women with families with 5 or more children is more than the national average (> 0.10), 0 otherwise
Distance to any health facility	Binary variable where 1 indicates woman lives in a county in which the average distance to any health facility is less than 33 min (the national median based on the sample), 0 otherwise
Quality of health facility	Binary variable where 1 indicates that woman lives in a county in which the proportion of women with at least one health facility with a good quality rating is more than 0.58 (the national average based on the sample), 0 otherwise
Female economic empowerment norm	Binary variable where 1 indicates woman lives in a county in which the proportion of women who work at job with a wage or salary is more than 0.10 (the national median based on the sample), 0 otherwise
*Individual*	
Age	Categorical variables. Indicator variable for whether respondent’s age lies in any of the following 10 year range age groups: 15–24; 25–34; 35–49.
Parity	Categorical variable. Indicator variable for whether the total number of children borne by respondent is in any of the following categories: 1 child; 2-4 children; 5 or more children
Woman’s Education	Categorical variable. Indicator variable for whether the highest grade completed by respondent is in any of the following categories: No school, Primary, Secondary, Tertiary
Household Head’s Education	Categorical variable. Indicator variable for whether the highest grade completed by household head is in any of the following categories: No school, Primary, Secondary, Tertiary
Birth history	Binary variable where 1 indicates whether respondent has ever had a child die, 0 otherwise
Poor	Binary variable where 1 indicates woman lives in a household whose consumption expenditure falls below the poverty line in 05/06 prices, 0 otherwise
Marital status	Categorical variables. Indicator variable for whether the respondent’s marital status grade is in any of the following categories: Married monogamously, married polygamous, divorced/separated, widow, never married
Female economic empowerment	Binary variable where 1 indicates woman has a job that pays a wage or salary, 0 otherwise

In order to categorize the community variables, the proportion of women with each attribute in a county was calculated. Histograms were then used to assess the distribution of each variable. Where the distribution of the proportions was not symmetric, the median proportion was used as the cutoff for the categorization of the county. The mean proportion was used as the cutoff for variables for which the distribution was symmetric. For example, in categorizing a community’s educational category, first the proportion of women in the various educational levels was determined. Communities within which the proportion of women with more than a basic level of education was more than the national average were categorized as highly educated or otherwise if the proportion was below the national average. Mekonnen et al., (2015) use a similar categorization strategy [39].

### Statistical analysis

Multivariate multilevel models are the appropriate specification for the relationships examined in this study based on three reasons. First, the data collection process was conducted using a two-stage cluster sampling strategy. Secondly, the variables included in the model specification are at two levels. Some at the individual level and others aggregated to the county level. Individuals are nested within counties to examine the associations between the woman’s community context and her place of delivery. Based on these reasons, using an ordinary least squares model will be inappropriate because the assumption of independence of observations will be violated and the estimated standard errors will be incorrect. Lastly, multilevel models include random effects at the individual and county level to account for unobserved individual and county level factors. The relevant specification of the model is shown below:

$$ {\mathrm{fbd}}_{\mathrm{ij}}\ast ={\upbeta}_0+{\upbeta}_{10}{\mathrm{I}}_{\mathrm{ij}}+\dots +{\upbeta}_{1\mathrm{Y}}{\mathrm{I}}_{\mathrm{ij}}\kern0.5em +{\upbeta}_{20}{\mathrm{C}}_{\mathrm{j}}+\dots +{\upbeta}_{2\mathrm{z}}{\mathrm{C}}_{\mathrm{j}}+{\upmu}_{\mathrm{j}}+\kern0.5em \upvarepsilon \mathrm{ij} $$

where fbd_ij_ = 1 if fbd_ij_* > 0; 0 otherwise.

Where fbd_ij_* is the propensity that woman i in community j uses a health facility for delivery contingent on her propensity to use a facility for delivery surpassing a certain threshold, β_0_ is the intercept estimate, β_10_ through β_1Y_ are the fixed estimates of the individual level variables, β_20_ through β_2z_ are the fixed estimates of the community variables, μ_j_ is the peculiar deviation (random effect) of the intercept of each community, and ɛ_ij_ is the individual level error term. μ_j_ is normally distributed with a constant variance.

The model in eq. 1 above is estimated using the generalized linear latent and mixed model command (GLLAMM) given that the outcome variable, whether the pregnant woman delivered at a health facility or not, is binary [40]. The conditional distribution is binomial and the link function is logit. Adaptive quadrature was specified. Survey weights were used in the bivariate analysis. Intraclass correlation coefficient (ICC) is used to assess how facility-based delivery differed between counties. The ICC explains the proportion of variation in facility-based deliveries that can be explained at the county level. An ICC value close to 1 indicates there is little variation within the counties and most of the variation is between counties. Conversely, an ICC value close to 0 indicates that most of the variation is within the counties and hence explained by individual level variables. Variance Inflation Factor (VIF) is used to test multi-collinearity for all the explanatory variables.

## Results

Table 3 highlights the descriptive characteristics of the women in the sample. More than a third of the women delivered outside a health facility. Fifty-nine percent of the women lived in rural counties. In the sample, more than a third of the women had experienced the death of a child and less than a quarter had more than a primary level of education. A quarter of the women lived in households in which household heads, who are usually male in this setting, had more than a primary level of education. Majority of the women were married monogamously and did not work for a wage. The mean time across the communities to any health facility from the village center was approximately 49 min.

**Table 3:** Descriptive characteristics of women in the sample

		Delivery in a health facility	
Variable	Number (%)	Yes	No
	n (%)	n (%)	n (%)
County Level			
Place of residence			
Rural	1962 (59.3)	1028 (52.4)	934 (47.6)
Urban	1348 (40.7)	983 (72.9)	365 (27.1)
Missing	0 (0.0)	0 (0.0)	0 (0.0)
Women who have had a child die in the past			
Yes	1859 (56.2)	1076 (57.88)	783 (42.12)
No	1426 (43.1)	927 (65.01)	499 (35.0)
Missing	0 (0.0)	8 (32.0)	17 (68.0)
Women with more than primary education			
Yes	1393 (42.1)	1002 (71.9)	391 (28.1)
No	1917 (57.9)	1009 (52.6)	908 (47.4)
Missing	0 (0.0)	0 (0.0)	0 (0.0)
Household head with more than primary education			
Yes	1421 (42.9)	1057 (74.4)	364 (25.6)
No	1889 (57.1)	954 (50.5)	935 (49.5)
Missing	0 (0.0)	0 (0.0)	0 (0.0)
Poverty concentration			
High	1800 (54.4)	898 (49.9)	902 (50.1)
Low	1510 (45.6)	1113 (73.7)	397 (26.3)
Missing	0 (0.0)	0 (0.0)	0 (0.0)
Prevalence of large family norm (% women with 5 or more children in sub-county)			
High	1775 (53.6)	1073 (60.5)	702 (39.6)
Low	1535 (46.4)	938 (61.1)	597 (38.9)
Missing	0 (0.0)	0 (0.0)	0 (0.0)
Time to any health facility from village center (minutes)			
High	1796 (54.3)	914 (50.9)	882 (49.1)
Low	1502 (45.4)	1094 (72.8)	408 (27.2)
Missing	12 (0.4)	3 (25.0)	9 (75.0)
Presence of at least one health facility with good quality rating			
High	1684 (50.9)	1113 (66.1)	571 (33.9)
Low	1599 (48.3)	881 (55.1)	718 (44.9)
Missing	27 (0.8)	17 (63.0)	10 (37.0)
Prevalence of female economic empowerment norm (Working women)			
High	1553 (46.9)	945 (60.9)	608 (39.2)
Low	1757 (53.1)	1066 (60.7)	691 (39.3)
Missing	0 (0.0)	0 (0.0)	0 (0.0)
Individual			
Age			
15–24	808 (24.4)	532 (65.8)	276 (34.2)
25–34	1549 (46.8)	980 (63.3)	569 (36.7)
35–49	947 (28.6)	496 (52.4)	451 (47.6)
Missing	6 (0.2)	3 (50.0)	3 (50.0)
Parity			
1	404 (12.2)	314 (77.7)	90 (22.3)
2–4	1262 (38.1)	834 (66.1)	428 (33.9)
> = 5	1644 (49.7)	863 (52.5)	781 (47.5)
Women’s Education			
No education	417 (12.6)	167 (40.1)	250 (60.0)
Primary	2001 (60.5)	1121 (56.0)	880 (44.0)
Secondary	532 (16.1)	442 (83.1)	90 (16.9)
Higher	80 (2.4)	73 (91.3)	7 (8.8)
Missing	280 (8.5)	208 (74.3)	72 (25.7)
Household Head’s Education			
No education	387 (11.7)	197 (50.9)	190 (49.1)
Primary	1718 (51.9)	946 (55.1)	772 (44.9)
Secondary	705 (21.3)	537 (76.2)	168 (23.8)
Higher	154 (4.7)	130 (84.4)	24 (15.6)
Missing	346 (10.5)	201 (58.1)	145 (41.9)
Ever had a child died			
Yes	1197 (36.2)	609 (50.9)	588 (49.1)
No	2109 (63.7)	1400 (66.4)	709 (33.6)
Missing	4 (0.1)	2 (50.0)	2 (50.0)
Poor			
Yes	1075 (32.5)	493 (45.9)	582 (54.1)
No	2228 (67.3)	1512 (67.9)	716 (32.1)
Missing	7 (0.2)	6 (85.7)	1 (14.3)
Marital Status			
Married monogamously	2062 (62.3)	1262 (61.3)	799 (38.8)
Married polygamous	681 (20.6)	376 (55.2)	305 (44.8)
Divorced/separated	270 (8.2)	186 (68.9)	84 (31.1)
Widow/widower	93 (2.8)	53 (57.0)	40 (43.0)
Never married	194 (5.9)	130 (67.0)	64 (33.0)
Missing	10 (0.3)	3 (30.0)	7 (70.0)
Female Economic empowerment (working for wage etc.)			
Yes	442 (13.4)	264 (60.0)	178 (40.3)
No	2845 (86.0)	1735 (61.0)	1110 (39.0)
Missing	23 (0.7)	12 (52.2)	11 (47.8)
N	3310		

Table 3 provides the percentage of respondents who delivered in a health facility by select characteristics of the counties in which they lived as well. For ease of interpretation, the counties were categorized as “low” or “high” dependent on whether the proportion was above or below the median or mean value from the distribution. Over two-thirds of urban residents delivered in a health facility compared to half of rural residents. Delivery in a health facility was most common amongst women who lived in communities that had few women who had previously lost a child. More women delivered at home in counties within which more women had less than a primary school level of education. Conversely, in counties categorized as high in terms of the proportion of household heads with secondary level or more education more women delivered in a health facility. Majority of women who lived in affluent counties delivered in health facilities. Lastly, counties with shorter average distance to a health facility and at least one facility with a good quality rating had more facility-based deliveries.

Table 4 shows the factors associated with facility-based delivery at both the individual and community levels. Column 1 uses a simple linear probability model to estimate the determinants of delivery in a health facility and to access the effect of multi-collinearity due to the two levels of data. The highest variance inflation factor of 5.78 is on the third category of the parity variable indicating that the woman has five or more children. The parity variable showed a high correlation (0.72) with the age variable. It was thus dropped in the subsequent analysis. The average variance inflation factor calculated from the model showed moderate correlation (1.97). This suggests that an appropriate model will be one that adequately accounts for the differing levels of data included in the model.

**Table 4:** Estimated odds ratios and confidence intervals from multiple regression models on the determinants of facility-based delivery in Uganda

Dependent Variable	Delivery in a health facility: OR (CI)^#^					
	OLS^+^	Empty Model	Mixed with individual level variables only	Mixed with community level variables only	Mixed with both levels of variables	Mixed without variables with most missing
	1	2	3	4	5	6
*Individual Level*						
Age (Ref: 15–24)						
25–34	0.074*** (0.021–0.128)		1.056 (0.820–1.359)		1.099 (0.852–1.417)	1.073 (0.860–1.339)
35–49	0.064* (− 0.003–0.131)		0.898 (0.673–1.200)		0.900 (0.673–1.204)	0.806* (0.624–1.041)
Parity (Ref: 1 child)						
2–4 children	− 0.146*** (− 0.215 - -0.076)					
5+ children	− 0.205*** (− 0.287- -0.123)					
Women’s Education (Ref: No school)						
Primary	0.085*** (0.030–0.141)		1.492*** (1.110–2.005)		1.436** (1.068–1.931)	
Secondary	0.197*** (0.125–0.268)		3.007*** (1.100–4.527)		2.831*** (1.874–4.276)	
Tertiary +	0.197*** (0.066–0.328)		4.529*** (1.545–13.273)		4.130*** (1.407–12.123)	
Household head’s Education (Ref: No school)						
Primary	0.014 (−0.045–0.072)		1.064 (0.772–1.467)		1.075 (0.779–1.483)	
Secondary	0.088*** (0.020–0.156)		1.743*** (1.192–2.548)		1.655 *** (1.129–2.427)	
Tertiary +	0.117** (0.017–0.218)		2.769*** (1.475–5.198)		2.614 *** (1.387–4.927)	
Marital Status (Ref: married monogamously)						
Married polygamous	−0.001 (− 0.045–0.043)		0.864 (0.679–1.100)		0.917 (0.719–1.168)	0.971 (0.781–1.208)
Divorced/separated	0.039 (−0.029–0.108)		1.310 (0.895–1.916)		1.285 (0.878–1.881)	1.219 (0.884–1.682)
Widow	− 0.020 (−0.123–0.083)		1.204 (0.682–2.126)		1.157 (0.647–2.070)	1.288 (0.759–2.188)
Never married	−0.121*** (− 0.212 - -0.030)		0.785 (0.488–1.263)		0.773 (0.480–1.244)	0.921 (0.624–1.361)
Number of children who have died	−0.027*** (− 0.045- -0.009)		0.867*** (0.788–0.954)		0.868*** (0.789–0.956)	0.829*** (0.760–0.905)
Female economic empowerment (Works for wage etc.)	0.031 (−0.020–0.083)		1.263 (0.946–1.687)		1.233 (0.922–1.649)	1.124 (0.870–1.452)
Socioeconomic Status (Poor)	−0.071*** (− 0.111- -0.031)		0.760*** (0.617–0.936)		0.776** (0.629–0.958)	0.602*** (0.500–0.726)
*County Level*						
High infant mortality county	−0.094*** (− 0.140- -0.048)			1.133 (0.762–1.685)	1.011 (0.673–1.518)	1.074 (0.728–1.584)
Highly educated women county	0.016 (−0.027–0.059			1.214 (0.786–1.876)	1.116 (0.717–1.737)	1.184 (0.773–1.812)
Highly educated household heads county	0.079*** (0.033–0.125)			1.957*** (1.265–3.028)	1.669 * (1.067–2.610)	1.960*** (1.277–3.009)
Poor county	−0.064*** (− 0.107- -0.021)			0.579*** (0.388–0.864)	0.642** (0.424–0.972)	0.663** (0.446–0.983)
Large family norm county (% women with more than 5 children in county)	0.109*** (0.065 - 0.153)					
Short distance to any health facility (minutes)	0.108*** (0.068–0.148)			1.642*** (1.108–2.433)	1.659*** (1.109–2.482)	1.675*** (1.139–2.463)
Good presence of at least one health facility with good quality rating	0.010 (−0.028–0.048)			1.299 (0.896 1.881)	1.222 (0.834–1.792)	1.273 (0.885–1.831)
Prevalence of economically empowered women (Working women)	−0.026 (− 0.064 - 0.012)			0.996 (0.679–1.461)	1.083 (0.730–1.605)	1.008 (0.692–1.468)
Rural	−0.030 (− 0.072–0.013)			0.759 (0.486–1.183)	0.858 (0.545–1.350)	0.826 (0.534–1.276)
Variance Inflation Factor (VIF)	1.970					
Proportional Change in Variance (PCV)			0.224	0.434	0.468	0.474
Intracluster Correlation Coefficient (ICC)		30.519	25.438	19.912	18.942	18.769
Log likelihood		− 1942.450	− 1512.475	− 1884.628	− 1467.053	− 1816.827
Random Effect Variance (Covariance)		1.445 (0.233)	1.122 (0.208)	0.818 (0.151)	0.769 (0.156)	0.760 (0.145)
Number of level 1 units	2643	3310	2688	3258	2643	3216
Number of level 2 units		141	141	138	138	138

Note: Level of significance: * signifies 0.1; ** signifies 0.05; *** signifies 0.01; + reports coefficients not odds ratios; #OLS – Ordinary Least Squares; OR – Odds ratio; CI – Confidence Interval

Column 2 presents the results from a null model that includes only the dependent variable and the cluster level aggregation. The null model shows the level of variation in facility-based delivery that is explained by factors at the county level. The intra-cluster correlation based on the null model estimate was 31%.

Column 3 through 6 present results from multilevel models that account for the different levels of data. In column 3 only individual level variables are included in the model. The intra-cluster correlation for this model is 25%. The more education a mother had the more likely she was to have given birth in a health facility. Similarly, women who lived in households with heads who had more than primary school education were more likely to have given birth in a health institution. Additionally, women who were poor were less likely to have given birth in a health facility. Lastly, the higher the number of the woman’s children who had died in the past the less likely the woman was to have delivered in a health facility. These relationships are all statistically significant.

Column 4 presents the model that includes only community level variables. The intra-cluster correlation for this model is 20%. These variables show some associations with the likelihood of delivery in a health facility. Women living in areas with high levels of poverty concentration are less likely to have delivered in a health facility. On the contrary, in counties with high concentration of women who live in households with household heads who have more than primary levels of education, there was increased likelihood that the women used a health facility for their most recent delivery in the last five years. Also, in counties where the average time it took to get to a health facility was short (less than 33 min) women were more likely to have delivered in a health facility.

Column 5 combines both the individual and community level factors in one model. In this combined model, the negative effect of poverty and previous adverse child outcomes at the individual level remains. The negative effect of county level socioeconomic status on the likelihood of delivery in a health facility remains as well. Between the two measures of educational attainment at the county only the proportion of heads of households with more than a primary level of education was statistically significantly associated with facility-based delivery. Communities with short travel time to health facilities were also positively associated with facility-based delivery.

Column 6 presents a model estimation excluding the variables with the most missing observations – individual level women’s education, household head education level. The key findings on county level household head’s educational attainment, socioeconomic status and travel time to a health facility remain robust in this restricted model specification.

## Discussion

This study is one of the few studies that explores the role that community factors can play, after controlling for household and individual level factors. It uses pooled data on women who have delivered in the last five years at the household-level to examine the community level determinants of facility-based delivery in Uganda. It also includes data from multiple years. The study findings support a positive association at the county level between facility-based delivery, education and access to health services, and a negative association between place of delivery and poverty. However, some of the findings were contrary to what was expected.

The most interesting finding was related to the association of the level of educational attainment of the household heads at the community level and a woman’s use of a facility for delivery compared to the level of educational attainment of the women themselves and their use of a health facility for delivery. The study findings revealed that individuals living in communities with a high level of education amongst the household heads were 1.67 times (95% CI: 1.07–2.61) more likely to have had a facility-based delivery compared to women living in communities where household heads did not have high levels of education. This finding is particularly poignant when contrasted with the insignificant association found between the proportion of women with more than a primary level of education in a county and facility-based delivery. The significance of the household head’s educational attainment compared to that of the mother herself in this study may highlight the important role played by other persons in ensuring that a pregnant woman delivers in a health facility. Additionally, in this particular context where household heads are most likely male, this finding may be capturing both the locus of the decision making as well as the awareness about issues that having a high level of education affords the head of the household.

Some evidence supporting positive associations between place of delivery and the available health supply market was found in the study. Although no association was found between individuals living in communities with at least one health facility with a perceived good quality rating, individuals living in communities with a short travel time to a health facility were more likely to have had an institutional delivery. Women who lived in counties with a short travel time (less than 33 min) were 1.66 times (95% CI: 1.11–2.48) more likely to have had a facility-based delivery compared to women who lived in counties with longer travel time to any health facility. Travel time has been found elsewhere to be an important determinant of place of delivery [10].

Furthermore, the study findings support a negative relationship between poverty levels and place of delivery. Women living in poor counties were only 0.64 times (95% CI: 0.42–0.97) as likely to have delivered in a health facility compared to pregnant women from communities with more affluent individuals. The economic status of the community may be a proxy for other characteristics of the community such as the availability and quality of health facilities in the community as well as the average educational attainment in the community. These variables are explicitly accounted for in our analysis. Other studies have documented the association between a mother’s wealth or socioeconomic standing and the likelihood that she had a facility-based delivery.

Some findings of the study are contrary to what is expected. For instance, no statistically significant association was found between paid working mothers and their use of health institutions for delivery. This finding contradicts the findings of a multi-country study inwhich female autonomy was found to have a significant association with the use of facility-based delivery assistance [13].

Furthermore, at the community level, the prevalence of child death was not associated with a facility-based delivery. This was an interesting measure to include in the model because in other studies previous adverse child birth outcomes has been included at the individual level and not at the community level as was done in this study. The study results showed significance only at the individual level. However, the association is inverse, which means that the more children a woman had lost to death previously the less likely they are to have delivered in a health facility. This suggests that at both an aggregate and individual level in this context there may still be a gap in knowledge, understanding, and awareness amongst mothers that biomedical assistance may improve survival for their offspring.

Overall, the intra-cluster correlation coefficient based on the null model indicated that approximately 31% of the variation in institutional delivery was due to variation in county attributes – observed and unobserved. The variation decreases when only individual level variables are included in the null model. This indicates that 22% of the variation across counties is attributed to compositional factors such as the women’s age, educational attainment of women and household heads, work status and marital status. On the other hand, when only community level variables are added to the null model the variation decreases by 43%. The decrease in variation when both individual and community level variables are included in the null model is 47%. Despite the reduction in variation with the inclusion of contextual (community) level variables, substantial variation across counties still remain which indicates there are contextual differences that are not captured by the county level variables included in this analysis.

Since 2010, the Ministry of health in Uganda has undertaken various projects aimed at improving the quality of care delivered in the health sector. The importance and prioritization of such efforts is reflected in the Health Sector Quality Improvement Framework and Strategic Plan (QIF&SP) 2015/16–2019/20 [41]. It is the common framework that guides all actors – health providers, policy makers, planners, programme managers and implementers, development partners - in the sector. These study results highlight individual preferences as strong determinants of whether pregnant women use health facilities for delivery and as recommended in the Lancet maternal health series call to action, prioritizing interventions aimed at ensuring that pregnant women receive respectful and supportive care when they utilize health facilities is one key approach to encouraging more deliveries in health facilities [8].

Limitations to this study include concerns about endogeneity related to selection. This is an issue that arises when individuals of a certain nature chose to live in neighborhoods with a certain profile so that the observed association might be driven internally as opposed to externally as espoused above. This challenge can be addressed using longitudinal data that allows the sequence of events in choosing residential location to be observed and controlled for in the study. Although this study finds associations of the various variables, the exact pathway or channels through which these factors influence individual level decision making is largely left unexplored. Another limitation pertains to the measurement error that may be ascribed to the nature of the birth history variable used. The question used to create this variable asked respondents how many of their children had died. It is not specifically restricted to babies so some of the children classified as dead maybe older than preferred for this particular study. Additionally, using community derived variables as the measure of social norms is not ideal. Nonetheless, according to available literature, using individual data to generate community level variables is ideal when there is additional information that can be gleaned from the aggregation of the variable that is not possible when the individual level variables are used [42]. However, if the number of units over which the characteristic is aggregated in the sample is small it may lead to inaccurate estimations.

## Conclusion

Findings from this study on community level factors that are associated with facility-based delivery showed limited evidence in broad support of the role of community level factors despite the findings in previous qualitative studies that contextual findings matter for promoting facility-based delivery. Nonetheless, the findings on household head’s education, community economic status and travel time to a health facility are useful for defining the attributes for targeting and developing relevant nation-wide community level health promotion campaigns. Future research may examine whether there is a herd effect with facility-based delivery when a community attains a certain threshold level of facility delivery use that makes the behavior accepted amongst pregnant women.

## Data Availability

The datasets analyzed for the current study are available in the World Bank Living Standards Measurement Study (LSMS) repository, [http://microdata.worldbank.org/index.php/catalog/lsms].

## Abbreviations

CI: Confidence Interval
GLLAMM: Generalized linear latent and mixed model
ICC: Intraclass correlation coefficient
LC1: Lowest administrative council area
UNPS: Uganda national panel survey
VIF: Variance inflation factor
